# Improving Spatial Adaptivity of Nonlocal Means in Low-Dosed CT Imaging Using Pointwise Fractal Dimension

**DOI:** 10.1155/2013/902143

**Published:** 2013-03-31

**Authors:** Xiuqing Zheng, Zhiwu Liao, Shaoxiang Hu, Ming Li, Jiliu Zhou

**Affiliations:** ^1^College of Computer Science, Sichuan University, No. 29 Jiuyanqiao Wangjiang Road, Chengdu 610064, Sichuan, China; ^2^School of Computer Science, Sichuan Normal University, No. 1819 Section 2 of Chenglong Road, Chengdu 610101, Sichuan, China; ^3^School of Automation Engineering, University of Electronic Science and Technology of China, No. 2006, Xiyuan Ave, West Hi-Tech Zone, Chengdu 611731, Sichuan, China; ^4^School of Information Science and Technology, East China Normal University, No. 500, Dong-Chuan Road, Shanghai 200241, China

## Abstract

NLMs is a state-of-art image denoising method; however, it sometimes oversmoothes anatomical features in low-dose CT (LDCT) imaging. In this paper, we propose a simple way to improve the spatial adaptivity (SA) of NLMs using pointwise fractal dimension (PWFD). Unlike existing fractal image dimensions that are computed on the whole images or blocks of images, the new PWFD, named pointwise box-counting dimension (PWBCD), is computed for each image pixel. PWBCD uses a fixed size local window centered at the considered image pixel to fit the different local structures of images. Then based on PWBCD, a new method that uses PWBCD to improve SA of NLMs directly is proposed. That is, PWBCD is combined with the weight of the difference between local comparison windows for NLMs. Smoothing results for test images and real sinograms show that PWBCD-NLMs with well-chosen parameters can preserve anatomical features better while suppressing the noises efficiently. In addition, PWBCD-NLMs also has better performance both in visual quality and peak signal to noise ratio (PSNR) than NLMs in LDCT imaging.

## 1. Introduction

Radiation exposure and associated risk of cancer for patients from CT examination have been increasing concerns in recent years. Thus minimizing the radiation exposure to patients has been one of the major efforts in modern clinical X-ray CT radiology [1–8]. However, the presentation of serious noise and many artifacts degrades the quality of low-dose CT images dramatically and decreases the accuracy of diagnosis dose. Although many strategies have been proposed to reduce their noise and artifacts [9–14], filtering noise from clinical scans is still a challenging task, since these scans contain artifacts and consist of many structures with different shape, size, and contrast, which should be preserved for making correct diagnosis.

Recently nonlocal means (NLMs) is proposed for improving the performance of classical adaptive denoising methods [15–17] and shows good performance even in low-dose CT (LDCT) imaging [18–20].

There are two novel ideas for NLMs. One is that the similar points should be found by comparing the difference between their local neighborhoods instead of by comparing their gray levels directly. Since gray levels of LDCT will be polluted seriously by noises and artifacts, finding similar points by local neighborhoods instead of by gray levels directly will help NLMs find correct similar points. The other important idea for NLMs is that the similar points should be searched in large windows to guarantee the reliability of estimation.

Following the previous discussion, the NLMs denoising should be performed in two windows: one is comparison patch and the other is searching window. The sizes of these two windows and the standard deviation *σ*_*r*_ of the Gaussian kernel, which is used for computing the distance between two neighborhoods, should be determined according to the standard deviation of noises [15–17], and these three parameters are identical in an image.

Some researchers find that identical sizes of two windows and identical Gaussian kernel *σ*_*r*_ in an image are not the best choice for image denoising [21–25]. The straightest motivation is that the parameters should be modified according to the different local structures of images. For example, the parameters near an edge should be different from parameters in a large smooth region.

An important work to improve the performance of NLMs is quasi-local means (QLMs) proposed by us [21, 22]. We argue that nonlocal searching windows are not necessary for most of image pixels. In fact, for points in smooth regions, which are the majority of image pixels, local searching windows are big enough, while for points near singularities, only the minority of image pixels, nonlocal search windows are necessary. Thus the method is named quasi-local whereit islocal for most of image pixels and nonlocal only for pixels near singularities. The searching windows for quasi-local means (QLMs) are variable for different local structures, and QLMs can get better singularity preservation in image denoising than classical NLMs.

Other important works about improving spatial adaptivity of NLMs are proposed very recently [23–25]. The starting point for these works is that the image pixels are parted into different groups using supervised learning or semisupervised learning and clustering. However, the learning and clustering will waste a lot of computation time and resource, which will hamper them to be applied in medical imaging. Thus we must propose a new method for improving the spatial adaptivity with a simple way.

In this paper we propose a simple and powerful method to improve spatial adaptivity for NLMs in LDCT imaging using pointwise fractal dimension (PWFD) where PWFD is computed pixel by pixel in a fixed-size window centered at the considering pixel. According to the new definition of PWFD, different local structures will be with different local fractal dimensions, for example, pixels near edge regions will be with relatively big PWFDs, while PWFDs of pixels in smooth regions will be zeros. Thus PWFD can provide local structure information for image denoising. After defined PWFD, which can fit different local structures of images well, we design a new weight function by combining the new PWFD difference between two considering pixels with the weight of original NLMs measured by gray level difference between two comparison windows. Thus using this new weight function, the proposed method will not only preserve the gray level adaptivity of NLMs but also improve the SA of NLMs.

The arrangement of this paper is as follows: In Section 2, the backgrounds are introduced, then the new proposed method is presented in Section 3, the experiment results are shown and discussed in Section 4, and the final part is the conclusions and acknowledgment.

## 2. Backgrounds

In this section, we will introduce related backgrounds of the proposed method.

### 2.1. Noise Models

Based on repeated phantom experiments, low-mA (or low-dose) CT calibrated projection data after logarithm transform were found to follow approximately a Gaussian distribution with an analytical formula between the sample mean and sample variance; that is, the noise is a signal-dependent Gaussian distribution [11].

The photon noise is due to the limited number of photons collected by the detector. For a given attenuating path in the imaged subject,  *N*_0_(*i*,  *α*) and *N*(*i*,  *α*) denote the incident and the penetrated photon numbers, respectively. Here, *i* denotes the index of detector channel or bin and *α* is the index of projection angle. In the presence of noises, the sinogram should be considered as a random process and the attenuating path is given by

(1) $$\begin{array}{c} r_{i}=-\ln\left[\frac{N\left(i,\;\alpha \right)}{N_{0}\left(i,\;\alpha \right)}\right], \end{array}$$

where *N*_0_(*i*,  *α*) is a constant and *N*(*i*,  *α*) is Poisson distribution with mean *N*.

Thus we have

(2) $$\begin{array}{c} N\left(i,\;\alpha \right)=N_{0}\left(i,\;\alpha \right)\exp\left(-r_{i}\right). \end{array}$$

Both its mean value and variance are *N*.

Gaussian distributions of ployenergetic systems were assumed based on limited theorem for high-flux levels and followed many repeated experiments in [11]. We have

(3) $$\begin{array}{c} \sigma_{i}^{2}\left(\mu_{i}\right)=f_{i}\exp\left(\frac{\mu_{i}}{\gamma }\right), \end{array}$$

where *μ*_*i*_ is the mean and *σ*_*i*_^2^ is the variance of the projection data at detector channel or bin *i*,  *γ* is a scaling parameter, and *f*_*i*_ is a parameter adaptive to different detector bins.

The most common conclusion for the relation between Poisson distribution and Gaussian distribution is that the photon count will obey Gaussian distribution for the case with large incident intensity and Poisson distribution with feeble intensity [11].

### 2.2. Nonlocal Means (NLMs)

Given a discrete noisy image *y*, the estimated value (${\hat{y}}_{i}$), for a pixel *i*, is computed as a weighted nonlocal average:

(4) $$\begin{array}{c} {\hat{y}}_{i}=\frac{1}{C\left(i\right)}\sum_{j\in B(i,\;r)}y_{j}\omega \left(i,j\right), \end{array}$$

where *B*(*i*, *r*) indicates a neighborhood centered at *i* and size (2*r* + 1)×(2*r* + 1), called searching window, and *C*(*i*) = ∑_*j*∈*B*(*i*, *r*)_ *ω*(*i*, *j*). The family of weights {*ω*(*i*, *j*)} depend on the similarity between the pixels *i* and *j* and satisfy 0 ≤ *ω*(*i*, *j*) ≤ 1 and ∑_*j*∈*B*(*i*, *r*)_ *ω*(*i*, *j*) = 1.

The similarity between two pixels *i* and *j*, *d* ^2^(*i*, *j*) depends on the similarity of the intensity gray level vectors *B*(*i*, *f*) and *B*(*j*, *f*), where *B*(*k*, *f*) denotes a square window with fixed size (2*f* + 1)×(2*f* + 1) and centered at a pixel *k*, named comparison patch:

(5) $$\begin{array}{c} d^{2}\left(i,j\right)=\frac{1}{{\left(2f+1\right)}^{2}}\sum_{k\in B(0,\;f)}{\left(y_{i+k}-y_{j+k}\right)}^{2}, \end{array}$$

and the weights *ω*(*i*, *j*) are computed as

(6) $$\begin{array}{c} \omega \left(i,j\right)=e^{-\max(d^{2}-2\sigma_{N}^{2},\;0)/h^{2}}, \end{array}$$

where *σ*_*N*_ denotes the standard deviation of the noise and *h* is the filtering parameter set depending on the value *σ*_*N*_.

### 2.3. Box-Counting Dimension

Box-counting dimension, also known as Minkowski dimension or Minkowski-Bouligand dimension, is a way of determining the fractal dimension of a set *S* in a Euclidean space *R* ^*n*^ or more generally in a metric space (*X*, *d*). To calculate this dimension for a fractal *S*, putting this fractal on an evenlyspaced grid and count how many boxes are required to cover the set. The box-counting dimension is calculated by seeing how this number changes as we make the grid finer by applying a box-counting algorithm.

Suppose that *N*(*ε*) is the number of boxes of side length *ε* required to cover the set. Then the box-counting dimension is defined as

(7) $$\begin{array}{c} \dim\left(S\right)=\underset{\varepsilon \to 0}{\lim}\frac{\log N\left(\varepsilon \right)}{\log\left(1/\varepsilon \right)}. \end{array}$$

Given an *N* × *N* image whose gray level is G, then the image is part into the *ε* × *ε* grids, which are related to *ε* × *ε* × *ε* cube grids. If for the *j*th grid, the greatest gray level is in the *ι*th box and the smallest is in the *κ*th box, then the box number for covering the grid is

(8) $$\begin{array}{c} n_{\varepsilon }=\iota -\kappa +1. \end{array}$$

Therefore the box number for covering the whole image is

(9) $$\begin{array}{c} N_{\varepsilon }=\sum_{j}n_{\varepsilon }\left(j\right). \end{array}$$

Selecting different scale *ε*, we can get related *N*_*ε*_. Thus we have a group of pairs (*ε*, *N*_*ε*_). The group can be fit with a line using least-squares fitting, the slope of the line is the box-counting dimension.

## 3. The New Method

In this section, we will present our new proposed algorithm in detail. The motivation for the proposed method is that SA of NLMs should be improved in a simpler way. The new PWFD is introduced firstly to adapt complex image local structures, and then the new weight functions based on PWFD are discussed. At the end of this section, the procedures of the proposed method are shown.

### 3.1. Pointwise Box-Counting Dimension

In image processing, the fractal dimension usually is used for characterizing roughness and self-similarity of images. However, most of works only focus on how to compute fractal dimensions for images or blocks of images [26–30]. Since fractal dimension can characterize roughness and self-similarity of images, it also can be used for characterizing the local structures of images by generalizing it to PWFD, which is computed pixel by pixel using a fixed-size window centered in the considered pixel. Thus, each pixel in an image has a PWFD and it equals the fractal dimension of the fixed-size window centered in the considered pixel.

Following the previous discussion, the pointwise box-counting dimension (PWBCD) starts from replacing each pixel *i* to a fixed-size window *r* × *r* centered at *i*. It is obvious that PWFD can be generalized to all definitions of fractal dimensions. However, in order to make our explanation more clearly, we only extend the new definition to PWBCD.

According to the new PWFD, PWBCD should be computed for each pixel in the image. For each pixel *i*, the PWBCD is computed in a fixed-size *r* × *r* window centered at *i*.

The *r* × *r* window is parted into the *ε* × *ε* grids, which are related to *ε* × *ε* × *ε* cube grids. If for the *j*th grid, the greatest gray level is in the *ι*th box and the smallest is in the *κ*th box, then the box number for covering the grid is

(10) $$\begin{array}{c} n_{\varepsilon }\left(i\right)=\iota -\kappa +1. \end{array}$$

Therefore the box number for covering the whole *r* × *r* window is

(11) $$\begin{array}{c} N_{\varepsilon }\left(i\right)=\sum_{j}n_{\varepsilon }\left(j\right). \end{array}$$

Selecting different scale *ε*, we can get related *N*_*ε*_(*i*). Thus we have a group of pairs (*ε*,  *N*_*ε*_(*i*)). The group can be fit with a line using least-squares fitting; the slope *k*(*i*) of the line is the box-counting dimension.

Note that each pixel in an image has a PWBCD value. Thus we can test the rationality for PWBCD by showing PWBCD values using an image. In these PWBCD images, high PWBCD values are shown as white points, while low PWBCD values are shown as gray or black points. If PWBCD images are similar to the original images with big PWBCD values near singularities and small PWBCD values in smooth regions, the rationality is testified.

**Figure 1:**
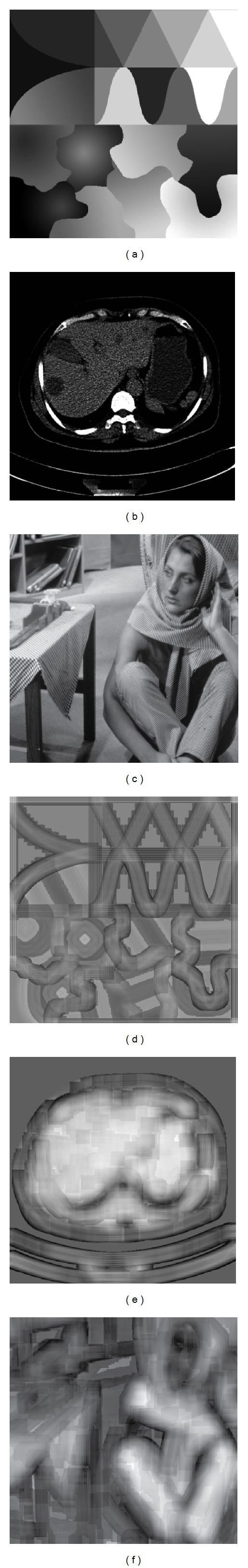
Images and their pointwise box-counting dimension images: the first row shows images while the second row shows their pointwise box-counting dimension images. Here *r* = 32 and *ε* = 2,  4,  8,  16,  32.

Figure 1 shows PWBCD images for three images: an test image composed by some blocks with different gray levels, a LDCT image, and 512 × 512 barbara. The white points signify the pixels with big fractal dimensions, while black points signify the pixels with small fractal dimensions. Here, *r* = 32 and *ε* = 2,  4,  8,  16,  32. Note that the white parts correspond the texture parts of barbara and soft tissues of the second image in the first row. Moreover, the PWBCD images are very similar to the original images which demonstrate that the PWBCD can be used for characterizing the local structure of images.

### 3.2. The New Weight Function

After defining the PWBCD, we must find an efficient and powerful way to use the PWBCD in NLMs directly. Just as discussed in the previous subsection, PWBCD can characterize the local structures for images well. Thus PWBCD should be used to weight the points in the searching patch. That is, (6) should be changed as

(12) $$\begin{array}{c} \omega \left(i,\;j\right)=e^{-\max\left(d^{2}-2\sigma_{N}^{2},\;0\right)/h_{1}^{2}-{\left(k(i)-k(j)\right)}^{2}/h_{2}^{2}}, \end{array}$$

where *k*(·) is FDBCD value for the considering pixel and is computed according to the method proposed in Section 3.1, *σ*_*N*_ denotes the standard deviation of the noise, *h*_1_,  *h*_2_ are the filtering parameters. *d* ^2^(*i*, *j*) is the similarity between two pixels *i* and *j* depending on the similarity of the intensity gray level vectors *B*(*i*, *f*) and *B*(*j*, *f*), where *B*(*k*, *f*) denotes a square window with fixed size (2*f* + 1) × (2*f* + 1) and centered at a pixel *k*:

(13) $$\begin{array}{c} d^{2}\left(i,j\right)=\frac{1}{{\left(2f+1\right)}^{2}}\sum_{k\in B(0,\;f)}{\left(y_{i+k}-y_{j+k}\right)}^{2}. \end{array}$$

Given a discrete noisy image *y*, the estimated value (${\hat{y}}_{i}$), for a pixel *i* is computed as a weighted nonlocal average:

(14) $$\begin{array}{c} {\hat{y}}_{i}=\frac{1}{C\left(i\right)}\sum_{j\in B(i,\;r)}y_{j}\omega \left(i,j\right), \end{array}$$

where *B*(*i*,  *r*) indicates a neighborhood centered at *i* and size (2*r* + 1)×(2*r* + 1), called searching window, and *C*(*i*) = ∑_*j*∈*B*(*i*, *r*)_ *ω*(*i*, *j*). Note that the family of weights {*ω*(*i*, *j*)} depend on the similarity between the pixels *i* and *j* and satisfy 0 ≤ *ω*(*i*, *j*) ≤ 1 and ∑_*j*∈*B*(*i*, *r*)_ *ω*(*i*, *j*) = 1.

### 3.3. The Steps of the New Method

The steps of PWBCD-NLMs are as follows.

1. Compute pointwise box-counting dimension for each ofthe pixels. For each of the pixels,given *r* = 2^*n*^,  *n* ∈ *Z* and *ε* = 2,  4,  …,  *r*, compute PWBCD according to Section 3.1, and get a matrix *K* with the same size as the image.
2. Compute weights. determine parameters: *σ*_*N*_,  *h*_1_, *h*_2_, the size of comparison window *cr*, and the size of the searching patch *sr*. Compute the difference between two comparison windows, *d* ^2^, using (13).Compute the weights *ω*(*i*, *j*) using (12).
3. Estimate real gray levels: estimate real levels $\hat{y}(i)$ using (14).

## 4. Experiments and Discussion

The main objective for smoothing LDCT images is to delete the noise while preserving anatomy features for the images.

In order to show the performance of PWBCD-NLMs, a 2-dimensional 512 × 512 test phantom is shown in Figure 1(a). The number of bins per view is 888 with 984 views evenly spanned on a circular orbit of 360°. The detector arrays are on an arc concentric to the X-ray source with a distance of 949.075 mm. The distance from the rotation center to the X-ray source is 541 mm. The detector cell spacing is 1.0239 mm.

The LDCT projection data (sinogram) is simulated by adding Gaussian-dependent noise (GDN) whose analytic form between its mean and variance has been shown in (3) with *f*_*i*_ = 2.5,  3.5,  4.0 and *T* = 2*e* + 4. The projection data is reconstructed by standard Filtered Back Projection (FBP). Since both the original projection data and sinogram have been provided, the evaluation is based on peak signal to noise ration (PSNR) between the ideal reconstructed image and reconstructed image.

The PWBCDs for images are computed according to Section 3.1, and the parameters are *r* = 32 and *ε* = 2,  4,  8,  16,  32. The new proposed method is compared with NLMs, and their common parameters includes the standard deviation of noise *σ*_*N*_ = 15; the size of comparison window is 7 × 7  (*cr* = 7), while the size of searching patch is 21 × 21  (*sr* = 21). The other parameter for NLMswhickis the Gaussian kernel for weights defined on (13) is *h* = 12 and the parameters for the new method are the sizes of Gaussian kernel for two weights defined on (12):  *h*_1_ = 15 for the weights of difference between comparison window and *h*_2_ = 10 for the weights between two PWBCDs. All parameters are chosen by hand with many experiments, which has the best performance.

Table 1 summarized PSNR between the ideal reconstructed image and filtered reconstructed image. The PWBCD-NLMs has better performance in different noise levels in the term of PSNR than NLMs.

**Figure 2:**
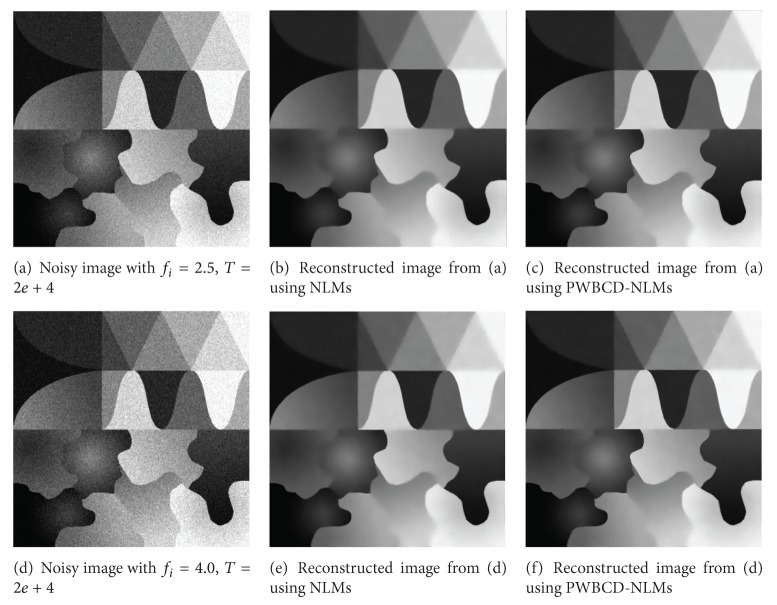
Noisy test images and reconstructed images.

Figure 2 shows noisy test images and their reconstructed images using NLMs and the proposed method. Although the reconstructed images are very similar to each other, the reconstructed images using the new method also show better performance in edge preservation especially in weak and curve edge preserving than the NLMs. Since PWBCD-NLMs provides a more flexible way for handling different local image structures, it has much good performance in denoising while preserving structures.

One abdominal CT images of a 62-year-old woman were scanned from a 16 multidetector row CT unit (Somatom Sensation 16; Siemens Medical Solutions) using 120 kVp and 5 mm slice thickness. Other remaining scanning parameters are gantry rotation time, 0.5 second; detector configuration (number of detector rows section thickness), 16 × 1.5 mm; table feed per gantry rotation, 24 mm; pitch, 1 : 1; and reconstruction method, Filtered Back Projection (FBP) algorithm with the soft-tissue convolution kernel “B30f”. Different CT doses were controlled by using two different fixed tube currents 60 mAs for LDCT and 150 mAs (60 mA or 300 mAs) for SDCT, resp.). The CT dose index volumes (CTDIvol) for LDCT images and SDCT images are in positive linear correlation to the tube current and are calculated to be approximately ranged between 15.32 mGy and 3.16 mGy [18].

On sinogram space, the PWBCDs for images are computed according to Section 3.1 and the parameters are *r* = 32 and *ε* = 2,  4,  8,  16,  32. The new proposed method is compared with NLMs and their common parameters includes the standard deviation of noise *σ*_*N*_ = 15; the size of comparison window is 7 × 7 (*cr* = 7), while the size of searching patch is 21 × 21  (*sr* = 21). The other parameter for NLMswhichis the Gaussian kernel for weights defined on (13) is *h* = 12 and the parameters for the new method are the sizes of Gaussian kernel for two weights defined on (12): *h*_1_ = 15 for the weights of difference between comparison window and *h*_2_ = 10 for the weights between two PWBCDs.

**Figure 3:**
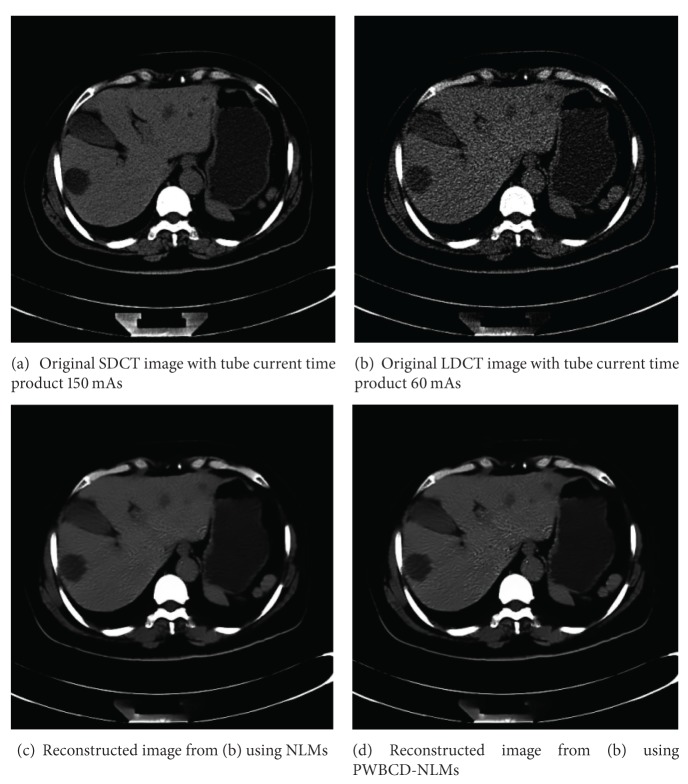
(b) Real LDCT reconstructed image, (a) related SDCT reconstructed images and (c)-(d) reconstructed images from LDCT sinogram using NLMs and the new method.

Comparing the original SDCT images and LDCT images in Figure 3, we found that the LDCT images were severely degraded by nonstationary noise and streak artifacts. In Figure 3(d), for the proposed approach, experiments obtain more smooth images. Both in Figures 3(c) and 3(d), we can observe better noise/artifacts suppression and edge preservation than the LDCT image. Especially, compared to their corresponding original SDCT images, the fine features representing the hepatic cyst were well restored by using the proposed method. We can observe that the noise grains and artifacts were significantly reduced for the NLMs and PWBCD-NLMs processed LDCT images with suitable parameters both in Figures 3(c) and 3(d). The fine anatomical/pathological features can be well preserved compared to the original SDCT images (Figure 3(a)) under standard dose conditions.

## 5. Conclusions

In this paper, we propose a new PWBCD-NLMs method for LDCT imaging based on pointwise boxing-counting dimension and its new weight function. Since PWBCD can characterize the local structures of image well and also can be combined with NLMs easily, it provides a more flexible way to balance the noise reduction and anatomical details preservation. Smoothing results for phantoms and real sinograms show that PWBCD-NLMs with suitable parameters has good performance in visual quality and PSNR.

## Figures and Tables

**Table 1:** PSNR for the test image.

Noise	PSNR of	PSNR of	PSNR of
parameters	the noisy image	NLMs	PWBCD-NLMs
*f*_*i*_ = 2.5, *T* = 2*e* + 4	23.29	34.19	** 34.95 **
*f*_*i*_ = 3.5, *T* = 2*e* + 4	21.88	33.79	** 34.59 **
*f*_*i*_ = 4, *T* = 2*e* + 4	21.30	33.45	** 34.16 **
